# Updates to the CASP Infrastructure in 2024

**DOI:** 10.1002/prot.70042

**Published:** 2025-09-01

**Authors:** Andriy Kryshtafovych, Maciej Milostan, Marc F. Lensink, Sameer Velankar, Alexandre M. J. J. Bonvin, John Moult, Krzysztof Fidelis

**Affiliations:** ^1^ Genome Center University of California, Davis Davis California USA; ^2^ Institute of Computing Science Poznan University of Technology Poznan Poland; ^3^ University of Lille, CNRS, UMR 8576—UGSF—Unité de Glycobiologie Structurale et Fonctionnelle Lille France; ^4^ Protein Data Bank in Europe European Molecular Biology Laboratory, European Bioinformatics Institute (EMBL‐EBI) Cambridge UK; ^5^ Bijvoet Centre for Biomolecular Research, Faculty of Science—Chemistry Utrecht University Utrecht the Netherlands; ^6^ Institute for Bioscience and Biotechnology Research University of Maryland College Park Maryland USA; ^7^ Department of Cellular Biology and Molecular Genetics University of Maryland College Park Maryland USA

**Keywords:** 3D structure prediction, alternative conformations, CASP16, protein structure, protein–ligand complexes, RNA structure

## Abstract

CASP (critical assessment of structure prediction) conducts community experiments to determine the state of the art in calculating macromolecular structures. The CASP data management system is continually evolving to address the changing needs of the experiments. For CASP16, we expanded the infrastructure to enable data handling of newly introduced categories and fully support pilot categories introduced in CASP15. This technical note also documents the integration of the CASP and CAPRI (Critical Assessment of PRedicted Interactions) systems.

## 1 Introduction

**FIGURE 1:**
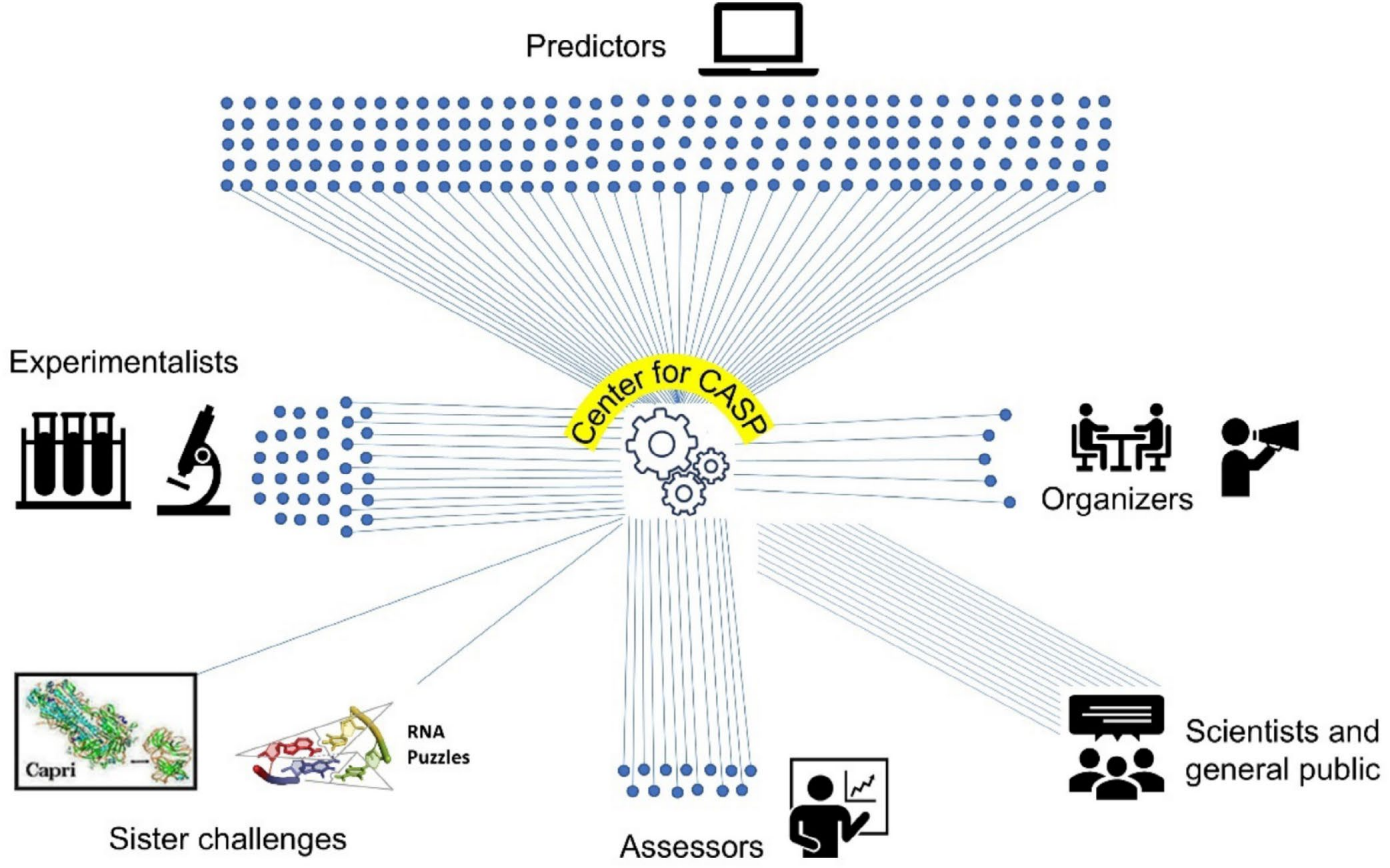
Center for CASP.

The critical assessment of structure prediction (CASP) drives progress in computational structural biology through large‐scale community experiments. Participants submit calculated structures that are evaluated using a range of metrics, and the results are assessed by independent scientists. The Center for CASP at UC Davis serves as the operational engine of CASP, providing the infrastructure for the experiments and handling all submitted and calculated data (Figure 1).

**FIGURE 2:**
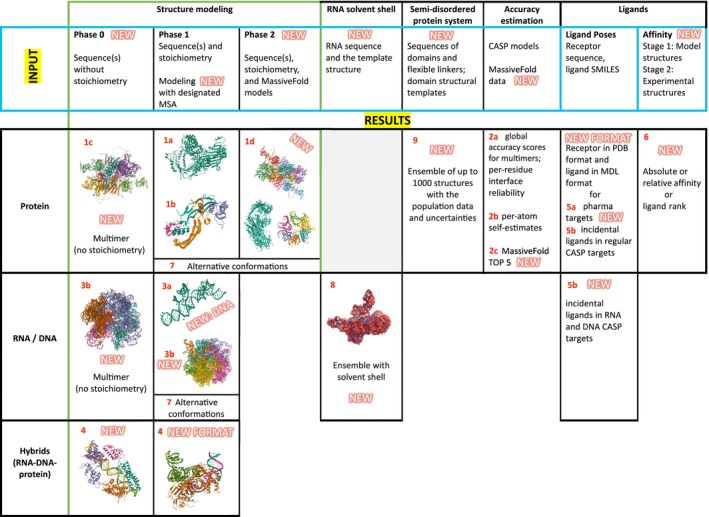
Scope of the CASP16 experiment. Top row: modeling categories; second row: information provided to modelers in each category; rows 3–5: expected modeling outcome for different types of macromolecules. Indexes in red (e.g., 1c) correspond to category numbers in the Introduction text. New developments are shown.

CASP has evolved from testing computational methods in one area—monomeric protein structures—to assessing methods for a range of computational tasks, including RNA, DNA, macromolecular complexes, and macromolecule/ligand complexes, as well as methods for estimating the accuracy of computed structures. The latest edition of CASP (CASP16, 2024) included nine broad modeling categories listed below and illustrated in Figure 2:

1. Protein structure:
  - Monomers [1].
  - Multimers [2].
  - Stoichiometry [2].
  - Modeling using large precomputed model sets (MassiveFold) [3].
2. Protein model accuracy:
  - Third‐party accuracy estimates of CASP models [4].
  - Per‐atom self‐estimates [4].
  - Third‐party estimates on large precomputed sets [3, 4].
3. Nucleic‐acid structure (RNA and DNA) [5]:
  - Monomers.
  - Multimers, with and without stoichiometry information.
4. Hybrid complexes (protein–RNA–DNA assemblies), with and without stoichiometry information [2, 5].
5. Structural ligand poses for protein– and RNA–ligand complexes [5, 6]:
  - Target sets from pharmaceutical companies.
  - Ligands present in general CASP structure targets.
6. Ligand binding affinities [6].
7. Multiple conformations of macromolecules [7].
8. Solvent spatial distribution around an RNA molecule [8].
9. Distribution of inter‐domain orientations for two protein domains connected by a flexible linker [9].

Some of these categories are well‐established CASP challenges (1a,b; 2a,b); some were introduced in the previous CASP (3a; 5b; 7). The remaining are new and require new processing pipelines (1c,d; 2c; 3b; 4; 5a; 6; 8; 9). This paper concentrates on the updates to the CASP infrastructure and describes the integration of CASP and CAPRI systems for seamless data flow between the two experiments.

## 2 CASP System

CASP software infrastructure consists of a set of frontend and backend services implemented mainly as CGI scripts, with an underlying PostgreSQL relational database management system for efficient processing, evaluating, and organizing submitted models. The dynamic content is served to the users, considering user roles and assigned privileges. The system integrates all CASP data and provides tools for:

- Participant registration.
- Target collection, verification, scheduling, and release.
- Checking consistency with model formats and acceptance deadlines.
- Notifying predictors of model formatting errors, and handling resubmissions.
- Model acceptance processing and storage.
- Model evaluation with multiple metrics.
- Results analysis.
- Communication with participants.
- Password‐protected access to anonymized evaluation results and confidential data for the assessors.
- Online publication of the results.
- Administrative management of CASP experiments.

In every CASP, elements of this highly interconnected system have to be adjusted to address the needs of new modeling categories and changes to existing ones. For the most recent CASP, changes to the system began with the organizers and assessors' review of existing evaluation measures. We eliminated measures that were too coarse‐grained for the current state‐of‐the‐art in structure modeling or rarely used by assessors in the last three CASPs. As a result, we reduced the number of evaluation tools in the existing categories from 30+ in CASP15 to 20 in CASP16. The new categories required designing formats and technical parameters for submissions, planning data workflows, and organizing data processing and evaluation pipelines. These are documented below.

### 2.1 Expansion of the CASP System to Integrate Evaluation for Categories Introduced in CASP15

The previous paper in this series [10] provided a detailed report on model formats, evaluation measures, and general principles for handling the three CASP15 pilot categories: RNA structure, ligand–protein complexes, and ensembles of alternative conformations. For these, the Center developed infrastructure for running the challenges and managed all the data, including models, targets, templates, and evaluation results. However, the evaluations themselves were performed by the assessment teams, who each explored different software packages and measures for assessment. The assessors provided evaluation recommendations for the current CASP experiment based on that CASP15 experience. These were incorporated into the CASP system in time for CASP16. As a result, all CASP16 evaluations for the pilot CASP15 categories were performed at the Center, saving the assessors time on setting up and running evaluation tools so they could concentrate on analyzing the results.

#### 2.1.1 RNA Targets

A systematic assessment of the accuracy of computed RNA structures was pioneered in 2010 by the RNA‐Puzzles team [11]. CASP collaboration with the RNA structure challenges began in the 2022 CASP15 [12]. There were 12 targets and 25 research groups testing 42 methods. In CASP16, the participation and number of targets increased, with 46 research groups running 65 methods on 35 targets. The larger number of targets and the growing interest resulted in a five‐fold increase in the amount of data, requiring a robust and comprehensive system for evaluation. With this in mind, we built an RNA evaluation system based on the well‐tested CASP protein evaluation system and incorporated the battery of measures already tested in CASP15. They are: local distance difference test scores (LDDT) [13], template modeling score (TM‐score) [14], global distance test total score (GDT‐TS) [15], root mean square deviation (RMSD) [16], and Clashscore [17]. The first four measures provide insight into the overall and local accuracy of models as compared to the reference structure, while the latter is a model‐only measure estimating stereo‐chemical plausibility of the coordinates. Two additional variants of these measures were also calculated, as requested by RNA assessors: an LDDT score without the stereo‐chemistry checks and a sequence‐independent version of the TM score (TM‐align). The newly updated OpenStructure software (version 2.8) [18] was integrated into the CASP evaluation pipeline and allows computation of the majority of the scores in a one‐stop manner. These scores are all adapted from protein structure evaluation. We also computed the RNA‐specific interaction network fidelity scores (INF) [19] for estimating the accuracy of canonical (Watson‐Crick, INF_WC), noncanonical (non‐Watson‐Crick, INF_nWC), stacking (INF_stacking), and all (INF_all) interactions between the nucleobases. These scores have long been used as accuracy measures in RNA‐Puzzles experiments and were also adopted for the assessment in CASP15. In CASP16, their calculation was included in the CASP evaluation system. Results of CASP16 RNA evaluations are presented in https://predictioncenter.org/casp16/results.cgi?tr_type=rna and discussed in [5].

#### 2.1.2 Ligand–Protein Complexes

Accurate computation of binding modes for organic ligands is of great practical relevance because of its role in rational drug design. There have been several previous challenge experiments in this area, most notably the D3R series [20], but all have ceased operation. In 2022, we revived systematic evaluation of ligand modeling by including ligand pose prediction in CASP [21], with 16 target proteins with accompanying ligands. There were eight such targets in CASP16. However, none of these are directly relevant to drug design. In CASP16, we secured four pharma target sets: three from Hoffmann‐La Roche (L1000, L2000, and L3000 target series) and one from Idorsia Pharmaceuticals (L4000 series). These sets included 233 individual protein–ligand complexes for ligand pose modeling. Thirty‐two research groups submitted models for these targets. As in CASP15, the assessment focused on ligands and their immediate surroundings. Two major evaluation measures used were the local distance difference test for protein–ligand interactions (LDDT‐PLI) and the Binding‐site superposed Symmetry‐corrected pose root‐mean‐square deviation (BiSyRMSD) [6, 18, 21]. The first measure estimates how well the native receptor–ligand interactions are reproduced, while the second evaluates the similarity of the model and reference ligand poses in terms of RMSD computed on the local superposition of binding sites. It should be noted that the CASP16 LDDT‐PLI score is a modified version of the corresponding CASP15 score, adjusted to properly penalize models for including erroneous non‐native ligand–protein contacts [18]. In addition to the receptor‐ligand measures, we also computed two binding site‐only measures: the LDDT‐lp measure comparing receptor's interatomic distances in binding pockets (lp for “ligand pocket”) and the corresponding BB‐RMSD measure (root‐mean‐square deviation of Cα atoms in the binding sites). All ligand accuracy scores were computed in‐house with the OpenStructure software package [18]. The results are reported at https://predictioncenter.org/casp16/results.cgi?view=targets&tr_type=ligand and discussed in [1, 5, 6].

#### 2.1.3 Ensembles of Alternative Conformations

The ability of macromolecules to adopt multiple conformational states is often related to their function. As such, the structural determination of alternative states is of big practical interest. However, this problem has not previously been included in CASP since until recently computational methods for determining single‐conformation structures were so imperfect that considering multi‐conformational nuances was impractical. With the accuracy of computed single structures reaching experimental levels in CASP14 and the increasing availability of experimental data on alternative conformations, in CASP15 we attempted to test the abilities of structure prediction methods in this area [22]. Technically, inclusion of alternative conformers in CASP did not require a new submission format as those were just more states of the 3D structures routinely processed in CASP. However, processing of submissions had to be modified. Different alternative conformations were either released as separate targets (e.g., T1249v1 and T1249v2) or the same target with the typical CASP five‐model allowance (e.g., R1203 or T1214). In both cases, all combinations of models and targets were considered and evaluated. All results were reported to the assessors, who analyzed them and developed a scoring system, combining evaluation scores for different variants into a single score estimating how well a group succeeded in predicting different conformations of the same target sequence [7].

### 2.2 Expansion of the System to Accommodate Novel CASP16 Categories

To address the evolving landscape of structural biology and the need to assess computational methods across a broader range of biological systems, the scope of CASP was significantly expanded in 2024; as shown in Figure 2 and briefly documented below.

#### 2.2.1 Stoichiometry Prediction and Phase 0 Structural Modeling of Complexes

In all previous CASPs, multimeric targets were released for prediction with the experimentally verified stoichiometry information. Each target was released only once, and all models submitted on a target were evaluated in one batch. In CASP16, we stepped away from this practice and implemented a two‐stage release procedure for selected multimeric structures. For targets where there was sufficient time available (as indicated by the target providers), sequences were first released without specifying the number of subunits within the complex, thus bringing the process closer to real‐life scenarios where multimeric composition is usually not known a priori. Then, after 2 weeks, we rereleased them with the stoichiometry information. The aim of this experiment was threefold: first, to encourage participants to develop approaches for predicting stoichiometry and to incorporate those in their structure modeling pipelines; second, to check what methods are already available and to test the ability of those to estimate the macromolecular composition of a target; and third, to compare the accuracy of models built with and without knowing the stoichiometry a priori.

The majority of protein multimeric targets (30 out of 40), nine multimeric RNA targets, and three hybrid protein–RNA–DNA complexes were put through the two‐stage release procedure. For more robust processing, we adopted the convention that the first number in a target name designates the modeling phase, and the following three numbers provide unique sequence(s) identifier. For example, T0206 is a phase‐0 target for structure 206, and T1206 is the phase‐1 target for the same target structure. Targets from different modeling stages were evaluated separately, and the CASP infrastructure was redesigned to report results of evaluations grouped by the target release stage—see “Phase 0” and “Phase 1” tabs in the results web pages, for example, https://predictioncenter.org/casp16/results.cgi?view=targets&tr_type=multimer.

*Note: the protein results pages also contain a “Phase 2” tab discussed in* Section 2.2.7.

Submitted models were evaluated in multimeric, monomeric, and evaluation unit (domains or combination of thereof) regimes. This pilot experiment revealed some interesting results [2, 23]; we plan to routinely include stoichiometry‐free challenges in the future CASPs.

#### 2.2.2 Multimeric RNA Structures

In the pilot CASP15 RNA structure prediction experiment, all targets were monomeric [12], and accordingly, we adopted technical specifications for models and templates, measures for evaluation, and principles for the organization of results already established for that type of target (see [10], and Section 2.1.1). In CASP16, several RNA structures were multimeric, and to accommodate these, we generalized target and model formats to allow submission of coordinates for multiple chains (Example 3 in https://predictioncenter.org/casp16/index.cgi?page=format). There were nine multimeric RNA targets in CASP16. All of them were released for prediction in two phases (see Section 2.2.1). Multichain submissions were split into separate subunits, and each was checked for format compliance and correspondence to the provided sequence. Similarly to proteins, multimeric RNA targets were evaluated both in monomeric and multimeric regimes. Overall accuracy of multimeric RNA models was evaluated with multimeric adaptations of monomeric scores (Section 2.1.1) as implemented in the OpenStructure suite [18]. The multimer‐specific evaluation concentrated on checking the accuracy in reproducing native interfaces. The OpenStructure software enabled calculation of the vast majority of interface‐specific scores that were used in the multimeric RNA assessment [5]: Precision reports the percentage of the correct interchain contacts among all interchain contacts in the model trimmed to the structurally resolved residues in the target; Recall enumerates the percentage of correctly reproduced native interchain contacts; the interface contact score (ICS) reports an *F*1 score representing the harmonic mean of the precision and recall in predicting interface contacts; the interface patch score (IPS) is a Jaccard coefficient showing similarity of the target and model interfaces; QS_scores (glob and best) are Jaccard coefficients showing similarity of target and model interface contacts, with the distinction that QS_glob accounts for all contacts in the model, while QS_best takes into consideration only residues structurally resolved in the target; iLDDT is the version of LDDT that considers only distances across interfaces, and the DockQ score [24] combines three measures used in CAPRI evaluation (LRMS, iRMS, and Fnat) [25]. Note that calculation of DockQ is problematic for complexes with a large number of subunits and residues as described in [18]. In CASP16, DockQ could not be calculated for two RNA and three hybrid complexes, all in excess of 1000 residues (R1251, R1254, M1268, M1271, M1297). Besides the above‐listed scores, we also report statistics on the submitted models: number of subunits, stoichiometry, number of contacts, and number of clashes. We designed new webpages for presenting results of multimeric RNA evaluation, for example, https://predictioncenter.org/casp16/results.cgi?view=targets&tr_type=rna_multi and https://predictioncenter.org/casp16/multimer_rna_results.cgi.

#### 2.2.3 DNA Structures

DNA is a new type of molecule in CASP. In CASP16, there was one monomeric DNA target and several DNA molecules as components of protein‐RNA–DNA complexes. Only submissions for the separate DNA target (D1273) were evaluated in the DNA track, while DNA components of hybrid complexes were evaluated from the perspective of their interactions with other assembly components.

Formats, submission procedures, and scripts for handling DNA data were based on those for RNA, adjusted to account for thymine (T) nucleobase in DNA instead of uracil (U) in RNA. Structures of the DNA models were evaluated with the same measures as RNA models. The OpenStructure evaluation resource successfully handled DNA data in calculating model‐to‐target structural similarity. However, we were not able to calculate INF‐type scores; their calculation required preliminary determination of interactions from the atomic coordinates, and the program that was used for this purpose in CASP15 and 16 (ClaRNA [26]) is RNA‐specific and not designed to work with DNA. Results of the DNA evaluation are reported together with the RNA results: https://predictioncenter.org/casp16/rna_results.cgi?target=D1273 and discussed in [5].

#### 2.2.4 Hybrid Multimolecular Complexes (i.e., Protein–RNA–DNA Assemblies)

For handling hybrid complexes, we combined already available CASP formats for protein, RNA, and DNA molecules. There were 16 different hybrid complexes in CASP16 (some released in two phases—see Section 2.2.1) and 56 groups submitted models for those. The biggest operational challenge was the model acceptance procedure, where we had to split multicomponent submissions into separate files, recognize the type of the molecule in each file, send those files to corresponding format verificators, and properly store the results. To simplify this task, we requested that protein chains in models and targets are named alphabetically (A, B, C,…), while RNA chains numerically (0, 1, 2,…). For evaluation of hybrid complexes, we used the same collection of evaluation measures as for multimeric RNAs (Section 2.2.2). Results for hybrid complexes are reported in https://predictioncenter.org/casp16/results.cgi?tr_type=hybrid and discussed in [2, 5].

#### 2.2.5 Protein–Ligand and RNA–Ligand Poses

Prediction of receptor–ligand poses saw two novelties in CASP16: first, we had incidental ligands with RNA structures, and second, large sets of ligand targets bound to the same protein. The data handling and evaluation procedures were built on those used in CASP15, with one significant technical change implemented to simplify submissions. In CASP15, we did not have a system that could handle both protein and ligand data in one file; thus, we requested separate submissions of protein and ligand portions of the complex. The receptor had to be in the PDB format, while the ligand in MDL format. Protein and ligand parts were sent to corresponding format verificators, and if correct, matched by target and model IDs, then joined. Obviously, not an ideal arrangement. For CASP16, we simplified the submission procedure and requested that both the receptor (protein or RNA) and ligand parts are submitted in the same file and in the same frame of reference. An example of a ligand pose prediction is provided in Example 6.1 on the CASP16 format page (https://predictioncenter.org/casp16/index.cgi?page=format). During the verification stage, the system automatically splits predictions and sends ligand and receptor to the appropriate model processing channels. Incidental and pharma ligands were evaluated with the same evaluation measures (Section 2.1.2), even though the evaluation workflows differ. For incidental ligands, models were checked at the submission, while for pharma targets, submissions were first stored without the format checks and verified only after the model collection deadlines. The reason for this difference is described further on (Section 2.2.10). OpenStructure successfully handled both protein‐ligand and RNA–ligand formats, and results of the evaluations are presented at: https://predictioncenter.org/casp16/results.cgi?view=targets&tr_type=ligand and reported in [1, 5, 6].

#### 2.2.6 Ligand Affinity

Predicting ligand affinity is crucial in drug discovery because it helps identify potential drug candidates and optimize drug design. Recognizing this, CASP16 included ligand affinity prediction. Data sets from Hoffmann‐La Roche contained information on the binding affinity for 140 protein–ligand complexes, including 17 in the chymase set (L1000) and 123 in the autotaxin set (L3000) [6, 27]; these were offered for prediction.

The affinity estimates were requested in one of the three forms: absolute affinity (i.e., the ligand dissociation constant *K*_d_), relative affinity (i.e., the ratio of each ligand's dissociation constant to that of an arbitrarily selected reference ligand), or ligand rank within each dataset. The affinity estimates could be submitted together with the ligand pose (Example 6.3 in https://predictioncenter.org/casp16/index.cgi?page=format) or alone (Example 6.2). In the former scenario, affinity estimates were separated from the rest of the submission (as described in Section 2.2.5) and stored in the form of affinity‐only submissions.

The ligand affinity prediction procedure had two stages: participants first submitted affinity estimates without being given the ligand‐protein structures, and then, after receiving that information, allowed structure‐based methods to be used. In the first stage, all 140 complexes with the binding affinity data were offered for prediction, and 28 groups participated. In the second stage, a subset of 110 targets with both structural and affinity data was released (17 chymase and 93 autotaxin targets), and 27 participants took part, including 19 who also participated in the first stage.

Accuracy of the affinity predictions in both stages was evaluated by the assessors [6]; and the summary of the results is presented in https://predictioncenter.org/casp16/zscores_ligand.cgi.

#### 2.2.7 Modeling Assisted by a Large Set of Possible Structures (Phase 2 Prediction Based on the MassiveFold Data)

In this experiment (the so‐called “Phase 2” prediction), we wanted to test if the availability of a large pool of AlphaFold models can help improve the performance of CASP groups either by providing starting templates from which to develop improved models, or by simply picking better models from the pool. After completing Phase 1 Modeling, CASP and CAPRI participants were provided with the results of extensive structural sampling for the target: thousands of models (typically 8040) computed with different AlphaFold protocols were made available to the participants. Computation of such a large set of models in a matter of several days was a formidable task requiring extensive computational resources not available at CASP, and we thank the MassiveFold group [3] for generating the data and allowing us to stage this experiment. Results of this Phase 2 prediction are discussed in the protein structure assessors' papers [1, 2].

#### 2.2.8 Identification of Best Models in Large Model Sets (MassiveFold)

This trial is related to the experiment described above (Section 2.2.7). Here, participants are asked to inspect MassiveFold model sets, identify models with the highest accuracy, and send a list of the top five models as described in Example 5C in https://predictioncenter.org/casp16/index.cgi?page=format.

Performance in identifying best models was evaluated by CASP16 model accuracy assessors [4]; the results of the evaluation are hosted on the CASP16 results website: https://predictioncenter.org/casp16/results.cgi?tr_type=accuracy&phase=1.

#### 2.2.9 Modeling Based on a Specific MSA

In CASP16 we introduced a challenge where predictors with MSA‐based methods were asked to generate an additional model based on the ColabFold MSAs [28]. Before the start of CASP16, we approached the ColabFold group and asked them to publicly post their models and MSAs of CASP protein targets after we closed the server prediction window (typically 3 days after target release). They provided such data for all phase‐1 targets (i.e., those with stoichiometry).

In this controlled prediction environment, we wanted to separate effects related to MSA construction from other methodological aspects of modeling approaches. ColabFold MSAs and models were provided through the ColabFold CASP16 pages: https://casp16.colabfold.com/colabfold_baseline. To differentiate models built on the ColabFold MSA from the others, we asked predictors to designate these models as “Model 6” (in addition to traditional CASP Models 1–5). Analysis of the results of the experiment is provided in the monomeric prediction assessment paper [1].

#### 2.2.10 Poses of Different Ligands Bound to the Same Protein

As described in Section 2.1.2, we obtained four datasets from pharmaceutical companies that included a total of 233 protein–ligand complexes for ligand pose modeling. This is a large number of targets. Coupling this with the fact that each predictor could submit five models per target, a single submission from a group could contain more than a thousand models, in total hundreds of megabytes of data.

In well‐established CASP categories, submitted models undergo immediate format and content verification as soon as a submission is received. For the pharma ligand pose prediction and two other new CASP16 modeling challenges (see Sections 2.2.11 and 2.2.12) where we also expected up to a thousand models per target, we could not do this because that much real‐time processing could clog the main CASP verification server, preventing acceptance of other incoming submissions. To solve this problem, in CASP16 we routed submissions with large data sizes to a different machine. Incoming sets of models had to be packaged (tarred) and uploaded through one of the designated gateways:

- https://predictioncenter.org/casp16/predictions_submission_LG.cgi for ligands,
- https://predictioncenter.org/casp16/predictions_submission_ENSMBL.cgi for semi‐disordered systems (Section 2.2.11), or
- https://predictioncenter.org/casp16/predictions_submission_WATER.cgi for solvent shell (Section 2.2.12).

After completion of model collection, the uploaded files are untarred and individual models are sent to appropriate format verificators.

In the ligand category, the results of format checks were inspected by the Center staff, and formatting errors were communicated to participants via email. Such a procedure requires extensive human involvement and is error prone due to multi‐versioning (resubmission) of predictions. For the future CASPs, we plan to upgrade the system to allow smoother automatic data handling. Ligand predictions that passed the verification stage were evaluated at the Center as described in Section 2.1.2. Results of the assessment are available in [6].

#### 2.2.11 Semi‐Disordered Protein System (“Protein A” Experiment, T1200, T1300)

This experiment was to model the structure of a two‐domain protein with a flexible linker. The structures of the domains were known, so participants were challenged to generate the range of conformations positioning the two domains relative to each other.

Two targets were released—one with the wild‐type linker (T1200) and another with a poly‐GLY linker (T1300). Linkers were 6‐residue long. For each of these, ensembles of models that represent a continuous distribution of interdomain orientation had to be packaged and submitted together with a file specifying the population and uncertainty of each ensemble member.

Collected predictions (https://predictioncenter.org/casp16/predictions_submission_ENSMBL.cgi) were sent to assessors; they were evaluated by comparison of calculated NMR RDC data with the experimental data and of the calculated SAXS data with the experimental curve. Results of the assessment can be found elsewhere in this issue [9].

#### 2.2.12 RNA Molecule With Solvent Shell (R1260)

In this challenge, we tested the capability of methods to build an ensemble of conformations for a ribozyme RNA with the solvent shell. For each conformation, predictors had to submit RNA coordinates together with at least a five Angstrom shell of water and ions. Participants were encouraged to submit up to 1000 system configurations (models), with a suggested minimum of 10 (https://predictioncenter.org/casp16/predictions_submission_WATER.cgi). After collection, submissions were transferred to the RNA assessors, who evaluated them by comparing the structure of the local solvent shell (water and ions) against cryo‐EM data. The assessment was performed on the assessors' side using tools locally developed by the assessment team. Results of the assessment and descriptions of the most interesting methods can be found in the RNA water shell assessment paper [8].

### 2.3 Integration of CAPRI and CASP Systems

CAPRI and CASP are organized as separate experiments, each with their own, albeit overlapping, community of participants. Integration of CASP and CAPRI infrastructures for common CASP/CAPRI rounds facilitates the participation of modelers in both experiments without duplication of efforts for the groups focused on modeling of macromolecular complexes. Furthermore, the two separate and somewhat different evaluations of the predictions are providing complementary views on biomolecular interaction prediction, with CAPRI implementing since the start the same community‐established assessment metrics, while CASP's assessment is more dynamic with the possible introduction of new quality assessment metrics, depending on the invited assessor group. The integration, therefore, brings significant benefits to the structure prediction community as a whole and advances our general understanding of biomolecular interaction.

#### 2.3.1 Target Preparation

Prior to offering a target for prediction to CAPRI participants, the target is evaluated for suitability by the CAPRI assessment team (Marc Lensink and Alexandre Bonvin), including considerations of prediction difficulty and likely stoichiometry. Nowadays, this amounts to running a default AlphaFold prediction. Acceptance of a target for prediction depends on the quality of the reference structure and the expected difficulty level. Low‐resolution structures are avoided, but there is no hard limit on the resolution as it is the quality of interfaces that guides the target selection (although 3.5 Å can be considered a soft limit). Target difficulty depends on many factors such as size, stoichiometry, number and types of molecules, expected conformational changes, and more, and we aimed at having a balance between easy and difficult targets. In the absence of a reference structure, the AlphaFold confidence level is used to assess the difficulty. Acceptance of a target then means setting up the CAPRI and CASP templates and target IDs. Typically, only sequence and stoichiometry information is provided. Note that the CAPRI template file contains a template chain for each chain in the system (even if the chains are identical), whereas the CASP template only contains a single chain per unique sequence in the target. This is because CAPRI does not participate in the Phase 0 stoichiometry prediction.

#### 2.3.2 Target Submission

**FIGURE 3:**
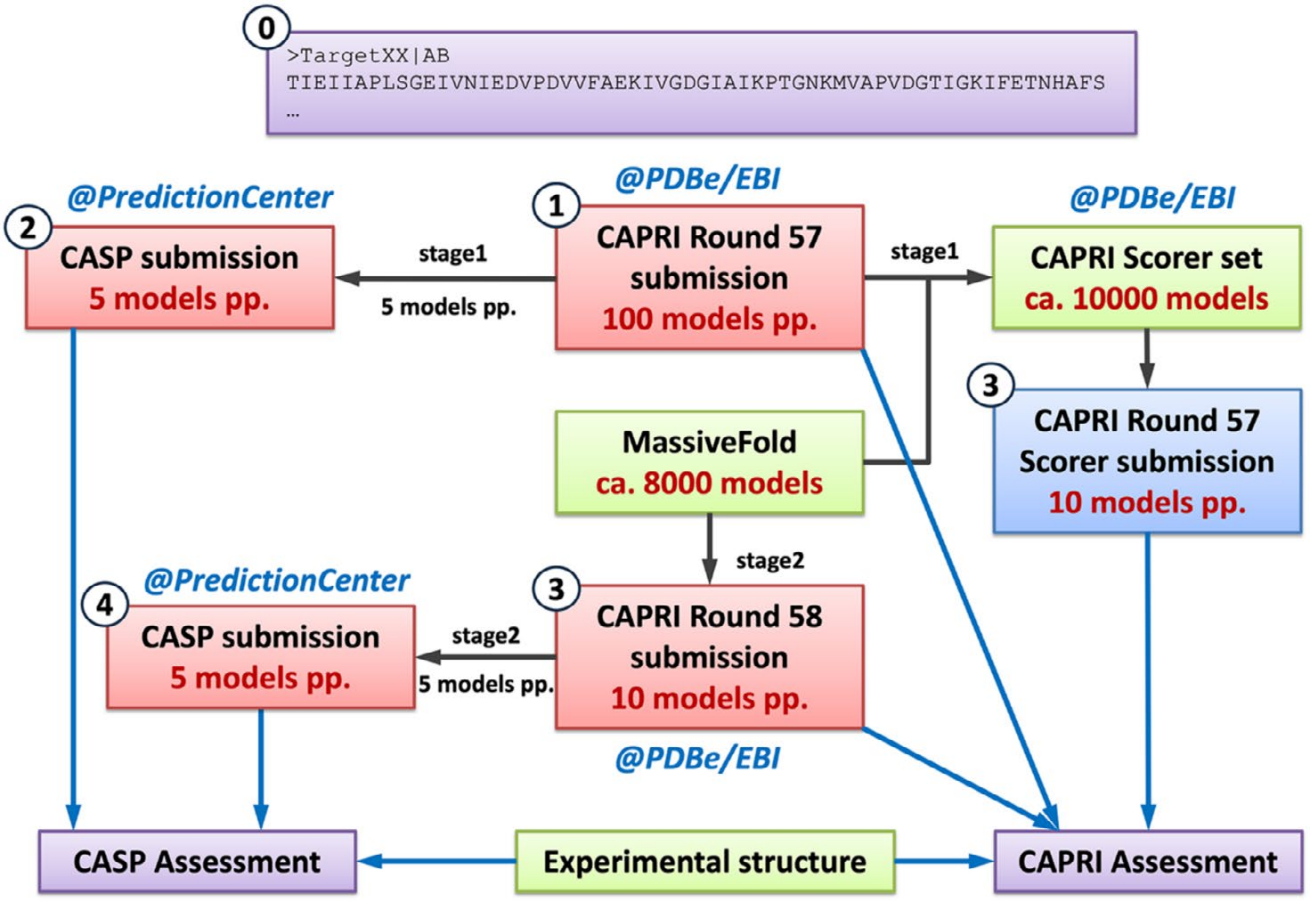
Flowchart of the CAPRI submission system. A round starts by providing the sequences of the components of the complex to be predicted to participants (Step 0). CAPRI Round 57 corresponds to CASP Phase‐1 prediction. The top five submissions to the CAPRI server (Step 1) are automatically forwarded to the CASP server (Step 2). Predictors models (up to 100) are then combined with the MassiveFold models to create the CAPRI score set. Scorers are then required to submit 10 models to the CAPRI server (Step 3). In parallel, the MassiveFold models are provided to the predictors for CAPRI Round 58, which corresponds to CASP Phase‐2 prediction. This round happens in parallel to the CAPRI Scoring Round and is not followed by a Scoring Round. The top five models of CAPRI Round 58 are automatically submitted to the CASP server (Step 4).

The CAPRI management and submission server is hosted by PDBe/EMBL‐EBI. Submission requires prior registration on the server. At the time of registration, the participants can register their CASP IDs, in which case submissions made on the CAPRI server will be automatically forwarded to CASP. This only works from CAPRI to CASP to avoid circularity and not vice versa. Figure 3 shows a flowchart of the CAPRI submission system for the latest CASP/CAPRI rounds.

In Round 57, from the CAPRI submission of at most 100 models per participant, the top five are automatically forwarded to the CASP submission system. During Phase 1, roughly 8000 MassiveFold models are produced; these are ranked by AlphaFold ranking confidence. Those models, together with up to 100 CAPRI models per participant, are shuffled and anonymized to enter the shuffled set for the CAPRI Scoring Experiment, which follows immediately after the regular prediction period (corresponding to CASP Phase 1 prediction). The scoring phase lasts at most one week. In parallel to the scoring in CAPRI, CASP Phase 2 predictions take place, where participants have access to the MassiveFold models. Also here, submissions made on the CAPRI server are forwarded to CASP. There is no scoring round for these submissions. Models from both prediction rounds and the scoring round are evaluated by both the CAPRI and CASP assessment protocols. As these differ, they provide slightly different but complementary views of the quality of the prediction.

#### 2.3.3 CAPRI Model Assessment

The CAPRI assessment is interface‐centric, follows the established CAPRI assessment criteria, and has been in use for many years [29, 30, 31]. Larger targets, or targets with multiple binding modes, may be separated into so‐called assessment units (AUs), which combine multiple interfaces. In such cases, the overall target assessment is either taken as the best of all AUs or their average values. A prediction is then characterized as either high quality, medium quality, or acceptable quality. All models not falling into any of these categories are deemed incorrect and are not included in the predictors' performance rankings.

## Author Contributions

**Andriy Kryshtafovych:** conceptualization, investigation, writing – original draft, methodology, visualization, writing – review and editing, software, project administration, data curation, supervision, resources, validation, formal analysis. **Maciej Milostan:** investigation, methodology, visualization, writing – review and editing, software, formal analysis, data curation, conceptualization, validation. **Marc F. Lensink:** visualization, methodology, writing – review and editing, software, resources. **Sameer Velankar:** methodology, visualization, writing – review and editing, software, resources. **Alexandre M. J. J. Bonvin:** methodology, visualization, writing – review and editing, software, resources. **John Moult:** conceptualization, investigation, validation, methodology, writing – review and editing, formal analysis, project administration, supervision, data curation. **Krzysztof Fidelis:** conceptualization, investigation, funding acquisition, methodology, validation, writing – review and editing, software, project administration, supervision, resources, data curation, formal analysis.

## Data Availability

All CASP16 data, including models, published targets, and evaluation results, are freely available through the Center for CASP website: https://predictioncenter.org. All evaluation software used in the analysis was freely downloaded from the Internet or obtained from the authors on request. The CAPRI models, together with their quality assessment, are publicly available as the CAPRI Scoreset, of which there are two releases so far [32, 33]. Following FAIR and open science principles, CAPRI plans to automate this process to make models and their metrics accessible as soon as each target is released in the PDB database.
